# The shifting impact and response to COVID-19 in Florida

**DOI:** 10.3389/fpubh.2024.1351690

**Published:** 2024-02-19

**Authors:** Scott A. Rivkees

**Affiliations:** Brown University School of Public Health, Providence, RI, United States

**Keywords:** pandemic, COVID-19, policy, vaccine, Florida, public health

## Abstract

The first cases of COVID-19 in Florida were diagnosed on March 1, 2020. Three years later, more than 7.3 million people have had COVID-19 in Florida, and more than 93,000 individuals have died from this illness. When considering the impact of COVID-19 on Florida, several key factors need to be considered, including that Florida was one of the most medically vulnerable states due to a substantial proportion of older individuals and those with underlying medical conditions. Florida also has a centralized Department of Health and Division of Emergency Management structure that facilitated response activities. Looking at the impact of COVID-19 on Florida, two distinct phases need to be considered: the pre-Delta variant phase from March 2020 to July 2021 and the Delta variant and beyond phase that began July 2021 and still continues. During the 16-month first phase, about 38,000 people died. Yet, 24,000 people died during the 5-months of the Delta variant wave from July to November 2021. During the Omicron waves that followed Delta, an additional 31,000 people died. Florida thus went from ranking 26th in death *per capita* in the United States at the end of the first phase to 10th a few months into the Delta wave and now ranks 8th. Why did these phases differ so dramatically in terms of mortality? During the first phase of the pandemic, adherence to established nonpharmacological and older adult protection measures was recommended. When COVID-19 vaccines became available in December 2020, there was an aggressive campaign to promote COVID-19 vaccination, and public acceptance was high. The second phase followed political opposition to CDC and public health expert guidelines, the rise of anti-vaccine sentiment and misinformation, and falling vaccination rates. These factors contributed to considerable population vulnerability to severe disease when the Delta variant hit. As the former State Surgeon General and Secretary of Health of Florida from June 2019 to September 2021, this report provides perspective on the shifting impact and response to COVID-19 in Florida, which is the third most populous state in the United States. This perspective shows the clear consequences of shifting from standard public health practices and vaccine promotion to attacks on public health and vaccines.

## Introduction

COVID-19 killed more than 1,100,000 people in the United States (1) and more than 93,000 people in Florida (2). More than 107 million people in the US and more than 7.5 million people in Florida have been documented to have COVID-19 infections, with the actual number of cases being far greater (2, 3).

In Florida, a Public Health Emergency (4) due to COVID-19 was issued on March 1, 2020 (5) and continued for 474 days (6). In response to COVID-19, there was a massive multiagency response in which Florida response agencies were allocated 8 billion dollars (7). The Florida Department of Health (DOH) and the Florida Division of Emergency Management (DEM) played leading roles during the response.

During the pandemic, states differed in population vulnerabilities, mitigation measures used, COVID-19 vaccination rates, and political approaches to the pandemic (8, 9). As such, state-to-state comparisons have attempted to relate COVID-19 outcomes to policies and actions (10–12). These analyses are complex, as mitigation strategies and implementation varied within states, and political strategies for COVID-19 control changed over time. Data from different pandemic phases are also grouped together in many studies, blending periods of low and high impact and masking the effects of changing policies and viral variants (8, 9, 11). In some studies, death rates have also been age-adjusted to facilitate comparison among different states; with this type of analysis, death rates seem lower in some states and higher in others than death *per capita* data (8, 9).

To assess COVID-19 impact, numbers of cases, hospitalizations, and deaths were collected and reported directly either by states, laboratories, hospital and nursing homes to the Centers for Disease Control and Prevention (CDC) or the Center for Medicare and Medicaid Services (CMS) during most of the Public Health Emergency (13). But with the advent of home testing, the varied expiration of state public health emergencies and reporting requirements, case and hospitalization data are often incomplete over the second half of the pandemic (12). Death data on the other hand, are still collected by the National Center for Health Statistics (14), and absolute numbers of deaths and death *per capita* data have been available since the start of the pandemic, making deaths *per capita* a practical comparator (13).

**Figure 1:**
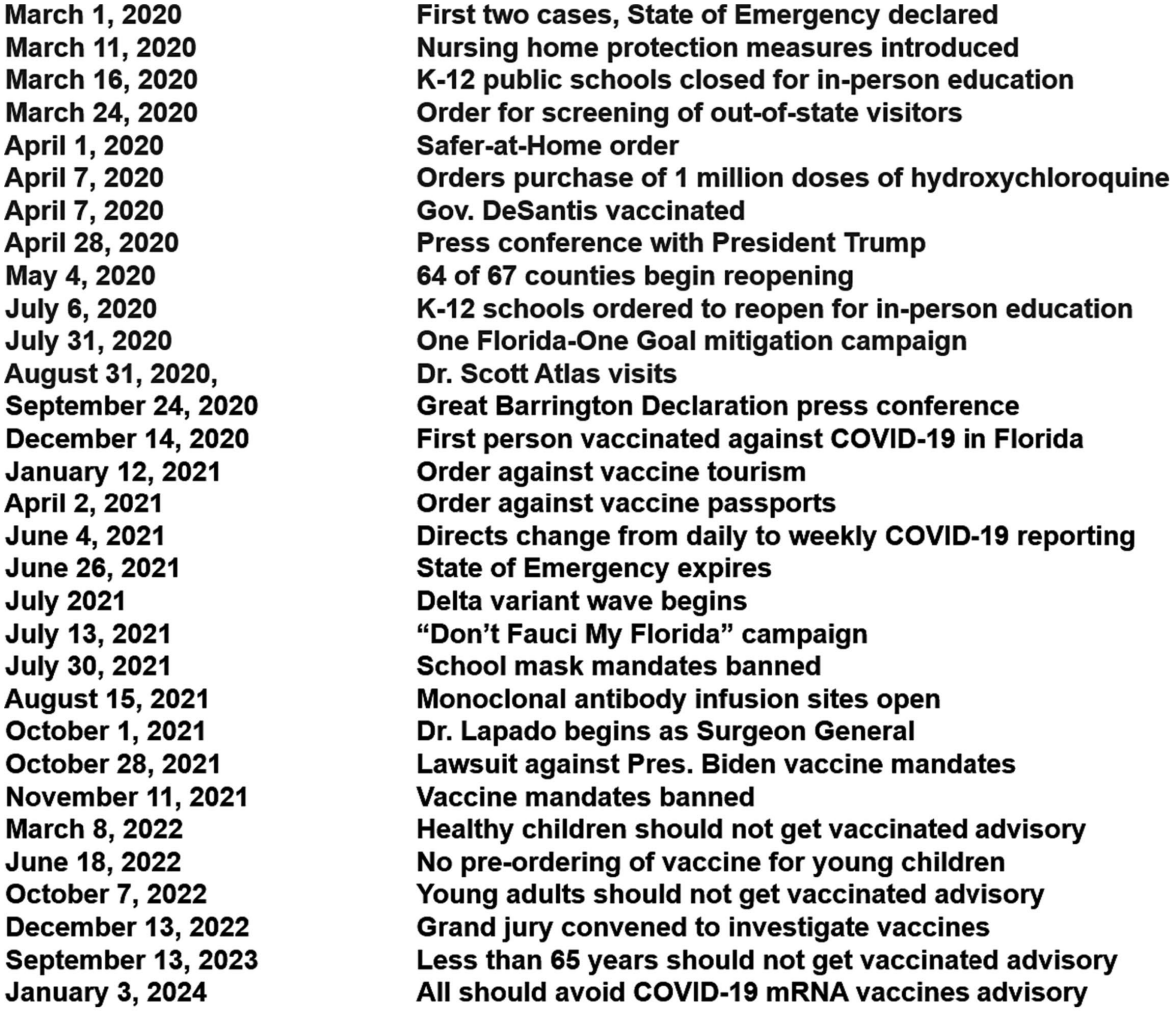
Key events in COVID-19 pandemic in Florida.

Florida is the third most populous state, with geographic and population diversity (15). Florida also had a changing political response over the pandemic (Table 1; Figure 1). Florida thus provides a special opportunity for assessing changes in policy, response, and COVID-19 variants within the same state.

**Table 1:** Chronology of key events during the COVID-19 pandemic in Florida.

Date	Key events in Florida	CDC and federal guidance	FL DOH guidance	Governor actions Executive orders
2020				
January 17		CDC briefing on novel coronavirus (16)		
January 21			FL DOH initiates STEPS plan (17)	
January 31		Monitoring of passengers arriving from China (18)		
February 28		FDA Approved COVID-19 tests (19)		
March 1	First 2 cases (20)		Public Health Emergency Declaration (4)	Executive order directs public health emergency (5)
March 7	First 2 Deaths (21)			
March 7				Emergency Operation Center opened (22)
March 11				Nursing home visitation suspended (23)
March 16				K-12 Schools close for in-person education (24)
March 16		Federal: 15 Days to Slow the Spread (25)		
March 17				Bars and restaurants closed (26)
March 25			Public health advisory issued (27)	
March 24				Airport screening of arriving passengers, highway roadblocks (28)
April 1				Safer-at-Home Order (29)
April 7				One million doses of hydroxychloroquine ordered (30)
April 16				
April 28				Gov. DeSantis Press conference at White House (31)
April 29				Reopening plan announced (32)
May 4	64 of 67 counties begin reopening (33)			
June 22			Updated Public Health Advisory (34)	
June 26				Bars reclosed (35)
July 21			Updated Public Health Advisory (36)	
July 31				One Florida-One Goal campaign (37)
July 6				Order for return in-person instruction (38)
August			FL DOH school support plan (39)	K-12 schools and universities reopen for in-person education (40)
August 31				Scott Atlas visits FL and hold press conferences (41)
September 15		CDC releases K-12 school COVID-19 guidelines (42)		
September 24				Press conference with Great Barrington Declaration originators (43)
September 25				Fines for not wearing masks prohibited (44)
December 11		Federal: FDA EUA approval for first mRNA vaccine (45) CDC issues recommendation for initial COVID-19 vaccines (46)		
December 14	First person vaccinated (47)			
December 23				State vaccine guidelines issued (48)
December 30	Cases per capita state rank: 24th (3) Deaths per capita state rank: 28th (3)			
2021				
January 21			Order against vaccine tourism (49)	
April 2				Vaccine passports banned (50)
April 7				DeSantis receives COVID-19 vaccine (51)
April 13		Pres. Obama and Biden host vaccination event (52)		
April 29			State employees to return for in-person work (53)	
June 4			Shift from daily to weekly COVID-19 reports (54)	
June 24	Condominium tower collapses (55)			
June 26				COVID-19 state of emergency expires (6)
June 30	Cases *per capita* state rank: 20th (3) Deaths *per capita* state rank: 26th (3)			
May 10		Vaccine approved for 12 years and older (56)		
July	Delta variant surge begins (57)			
July 13				Do not Fauci My Florida campaign (58)
July 30				Bans mask mandates in schools (59)
August 6			Rules for school year issued (40)	
August 15	Monoclonal antibody sites open (60)			Early treatment saves lives campaign (61)
October 1			Dr. Joseph Ladapo replaces Dr. Scott Rivkees as Surgeon General (62)	
October 28				Lawsuit against Biden vaccine mandates (63)
November 2		Vaccine approved for 5 years and older (56)		
November 11				Vaccine mandates banned (64)
December 30	Cases per capita state rank: 9th (3) Deaths *per capita* state rank: 10th (3)			
2022				
January	Omicron variant surge begins (65)			
January 14				Will not enforce mandatory vaccination for healthcare workers (66)
January 24				State closes monoclonal antibody sites (67)
March 8			Announces healthy children should not get vaccinated (68)	
June 18		Vaccine approved for 6 months and older (56)		
				Only state not to preorder vaccine for young children (69)
September 28	Hurricane Ian (70)			
October 7			Advisory against vaccinating young adult men (71)	
December 13				Grand jury to investigate vaccines (72) Public Health Integrity Committee announced (73)
December 30	Cases *per capita* state rank: 9th (3) Deaths *per capita* state rank: 10th (3)			
2023				
			Advisory against vaccinating those less than 65 years (74)	
December 30	Death pe capita state rank: 8th (3)			
2024				
January 3			Advisory against use of COVID-19 mRNA vaccines for all ages (75)	

**Figure 2:**
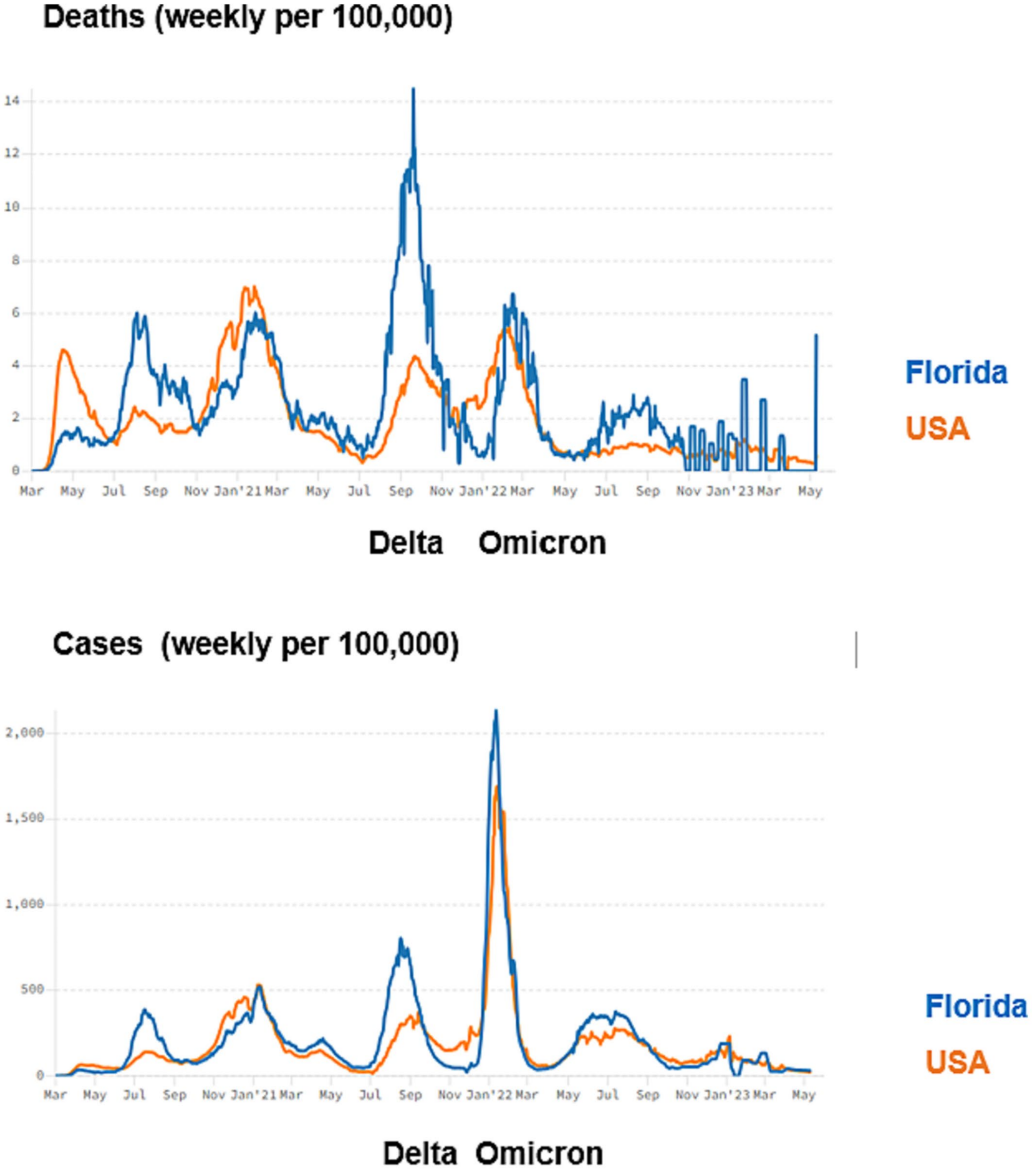
Deaths and Cases per 100,000 in Florida and United States over time. The period of the Delta and Omicron waves are shown.

In Florida, the pandemic can be split into two major phases: the pre-Delta variant phase from March 2020 to July 2021 and the Delta variant and beyond the stage that began July 2021 (Figure 2). During the first phase of the pandemic, the White Coronavirus Task Force and CDC recommendations were followed by the state, including physical distancing, crowd avoidance, vulnerable population protection, and face mask use (76). During this phase, the state locked down in April 2020, and state reopening began in early May 2020. In August 2020, public K-12 schools and universities opened for face-to-face activities. When it became available, an aggressive campaign to promote COVID-19 vaccination, especially in those who were older than 65 years (77). Reflecting the impact of these public health and vaccination measures, including 9 months before the COVID-19 vaccine became available, Florida ranked 20th and 26th among states in cases and deaths of COVID-19 *per capita* on June 30, 2021, before the Delta variant phase, respectively (78).

The second phase of COVID-19 in Florida followed rising political opposition to COVID-19 mitigation measures and vaccines that began in early 2021, resulting in substantial population vulnerability to COVID-19. The 5 months of the Delta variant wave from July through November 2021, took a brutal toll on Floridians. Almost two-thirds as many people died during this period as over the 16 months before, as Florida had one of the highest actual and *per capita* death rates in the country during this time. By the end of the Delta variant wave, Florida rose to 10th in cases and deaths *per capita*. Reflecting the fact that Florida COVID-19 death rates have continued in excess of the rest of the United States, Florida now ranks 8th in death *per capita* (3).

Examining the different phases of COVID-19 in Florida, there are many lessons to be learned. The focus of this report is to provide insights into the shifting impact of COVID-19 in Florida by detailing important events in the pandemic, the unique vulnerabilities of Florida, the state pandemic response structure and strategies, and the changes in the response to the pandemic by Governor DeSantis.

### Early events in the pandemic in Florida

Our first alert to COVID-19 was a message from the US Centers for Disease Control and Prevention (CDC) on January 8, 2020 (79), describing an outbreak of severe pneumonia of unknown cause in Wuhan, China. On January 17, 2020, the first public CDC briefing was held about COVID-19 (16). The first reported case of COVID-19 in the US was on January 20, 2020 (80).

In January 2020, DOH was dealing with a severe outbreak of hepatitis A and had an incident command team (IMT) that was active and redirected to prepare for COVID-19 (81). The DOH Division of Emergency Preparedness and Community Support team, which responds to disasters, was mobilized in mid-January for COVID-19. We also began engagement with the federal agency for Administration for Strategic Preparedness and Response (ASPR) with in-person meetings starting the third week of January (82).

On January 31, 2020, the federal government issued a travel advisory for individuals coming from China (18). Individuals arriving from China must isolate for 14 days and monitor symptoms. DOH was provided information about individuals arriving in Florida from China, and DOH staff contacted these individuals to begin at-home symptom monitoring. Of more than a 1000 individuals who came to Florida from China and were monitored, very few showed symptoms or tested positive for COVID-19.

On February 15, 2020, Vice President Pence, a White House Coronavirus Task Force member, visited Florida and held a press conference where the state of pandemic preparedness was discussed (83).

On February 28, 2020, the first day the CDC allowed state laboratories to use their internally developed COVID-19 test kit, we identified two individuals testing positive for COVID-19 (84). One was a woman who recently returned from northern Italy, a COVID-19 hotspot. The other was a man from Manatee County who had not traveled out of Florida but had recently visited the Florida Keys, where he was presumably exposed to a visitor with COVID-19.

The first deaths from COVID-19 in Florida occurred on March 7, 2020, in two older individuals who contracted COVID-19 during international travel (21).

Over the first half of March 2020, DOH contact tracing teams identified the source of exposure for all individuals who tested positive for COVID-19. Over the second half of the month, DOH could no longer find the potential sources of infection. Following CDC guidelines (85), we declared that there was community spread of COVID-19 in Florida.

In March 2020, national concern was that hospitals could be overwhelmed with COVID-19 patients. The United States Surgeon General recommended that hospitals limit elective procedures (86). On March 20, 2020, an executive order was issued in Florida limiting elective surgical procedures (87).

Ohio was the first state to close schools due to the COVID-19 pandemic on March 12, 2020 (88). The next day, the Florida Department of Education announced that Florida K-12 schools would close on March 16 (89). Florida state universities ceased face-to-face education in mid-March 2020 (90).

On March 16, 2020, the White House COVID-19 task force announced the “15 days to slow the spread” program (25). In mid-March 2020, counties and states issued stay-at-home or safer-at-home directives. South Florida counties issued orders limiting activities on March 17, 2020 (91). On April 1, 2020, the Governor issued Florida’s Safer-at-Home order (29), which remained in effect until phased reopening the following month.

The “Safe, Smart, Step-by-Step Plan for Florida’s Recovery” was the state’s reopening plan and was released on April 29, 2020 (32). On May 4, 2020, 64 of the 67 counties began the three-phase reopening plan (33). The three counties that did not initially reopen, Miami-Dade, Broward, and Palm Beach, accounted for more than 70% of the state’s cases at the time. These remaining counties began phased reopening 3 weeks later (92).

In August 2020, K-12 schools and the state university system reopened for face-to-face education as cases of COVID-19 increased during the summer (93).

COVID-19 vaccine first arrived in Florida December 14, 2020 (94), facilitating the shift from mitigation measures to vaccination as a primary strategy for COVID-19 disease control.

### State of Florida vulnerabilities

By the end of January 2020, data from China showed that COVID-19 was especially deadly for older individuals (95). As cases in the US climbed, CDC data also showed that the risk of dying from COVID-19 rose progressively in people older than 65 years (96). Individuals with underlying medical conditions, including respiratory illness, immunocompromised states, and obesity, were especially vulnerable to COVID-19 as well (97).

In Florida, more than 21% of the population is 65 years and older, making it second to Maine in older people *per capita* (98). Out of nearly 22 million people in the state, more than 4.5 million are 65 or older. Based on the numbers of those with underlying medical conditions, an additional 2 million individuals younger than 65 were projected to be especially vulnerable to COVID-19 (99).

The CDC created a COVID-19 vulnerability index that accounted for population age and risk factors by meshing these risk factors together (100). By this metric, Florida was one of the most vulnerable states for COVID-19 severe illness and death, as well.

Complicating the risk for the state, in 2020, there were over 4,000 licensed nursing homes and assisted living facilities with 150,000 residents (101). Showing how deadly the virus could be within these facilities, at the end of February 2020, the first outbreak of COVID-19 in a nursing home in the US in Kirkland, Washington, resulted in many deaths (102). Soon after, deadly outbreaks of COVID-19 were reported in nursing homes in New York (103) and New Jersey (104).

### Centralized response structure in Florida

Health departments nationwide have a centralized, decentralized, or hybrid organizational structure (105). A significant advantage of the Florida response was a centralized structure, with the DOH pivotal in directing statewide policy and activities at the county level. Florida has 67 counties, each with a county health department led by a county health officer (106). There are more than 12,000 employees of the DOH, including approximately 3,000 individuals working in Tallahassee, the state capital city.

DEM also has a centralized structure, interacting with county directors of emergency management. Because Florida has responded to many natural disasters and storms over many decades, the DEM is nationally renowned for its ability to respond to state emergencies.

DEM plays a significant role in providing logistical support during times of emergency. During the pandemic, DEM launched, orchestrated, and administered mass testing sites, mass vaccination sites, field hospitals, development of COVID-19-only nursing homes, movement of relief supplies around the state, and monoclonal antibody infusion sites. Former Florida House member Jared Moskowitz, now in the US Congress, was the DEM Director for most of the COVID-19 pandemic until April 2021, when DEM Deputy Director Kevin Guthrie succeeded him (107).

During the pandemic, the Agency for Health Care Administration (ACHA) monitored hospital responses and data collection and had authority over nursing homes. Over the first half of the pandemic, Sec. Mary Mayhew directed the agency (108).

During states of emergency, such as in hurricanes, operations are managed in the Emergency Operations Center (EOC) in Tallahassee. This command center is a hub for state agencies, emergency service function (ESF) teams, and federal partners, facilitating a coordinated state multiagency and federal response. To prevent COVID-19 outbreaks in the EOC that could jeopardize the response, symptom and COVID-19 contact screening were required upon entry, along with facemasks and twice-weekly COVID-19 testing.

### The Florida STEPS plan

The primary plan that guided the DOH response to COVID-19 was implemented in January 2020. This was labeled the Florida STEPS plan (1): Social distancing (2), Testing and contact tracing (3), Older adult and medically vulnerable population protection (4), Preparing hospitals for a patient surge and health care worker protection, and (5) Stopping the introduction of COVID-19 into the state (17). Implementation of these components involved interactions with county departments of health, other state agencies, and community partners. These recommendations were based upon guidelines issued by the White House Coronavirus Task Force, the CDC, the Food and Drug Administration (FDA), and CMS. Florida instituted many of these protective COVID-19 control measures before Democratic-lead states and kept them longer than most other Republican-lead states (8, 109).

*The DOH emphasized social distancing and nonpharmaceutical interventions* throughout the pandemic. On March 1, 2020, the declared Public Health Emergency highlighted the importance of mitigation measures, including physical distancing, symptom monitoring, and facemasks. The DOH and DEM distributed millions of facemasks throughout the state as part of the response. Public information campaigns, including the “One Florida-One Goal” campaign was launched in the summer of 2020 to encourage mitigation strategy adherence (37). Separate public health advisories were issued From March 25 through July 21, 2020, emphasizing these measures (34, 36, 110). Beginning in April 2020, the DOH had regular calls with the Florida Chamber of Commerce to discuss mitigation measures and the importance of indoor ventilation.

*Testing and contact tracing* were activities the DOH planned before the first case in the state. DOH laboratories prepared for molecular-based COVID-19 testing at the end of January 2020. When the CDC test kit was approved, testing at DOH state laboratories began on February 28, 2020.

When the first FDA emergency use approved (EUA) commercially available molecular COVID-19 test kit became available (111), the state purchased enough kits to perform more than 650,000 tests. This exceeded the number of all test kits purchased by laboratories in the rest of the United States. Faced with shortages of reagents and supplies to run these and other COVID-19 tests, DOH established a clearinghouse whereby reagents used for university research purposes were redistributed to laboratories where COVID-19 testing was performed.

During March 2020, we expanded public reporting of COVID-19 cases by developing a public dashboard in which county-level information was displayed (112). Personnel at county health departments performed contact tracing of individuals with or exposed to COVID-19. Beginning in February 2020, to provide additional epidemiological support, the DOH hired more than 600 students and faculty from public health schools in the state (113). By June 2020, more than 4,400 people were involved in COVID-19 contact tracing (114).

*Older adult protection* and the protection of those with underlying medical conditions were a significant focus for the state. When one looks at comparative mortality in Florida versus other states over the first year and a half of the pandemic (first phase), despite a substantial at-risk population, Florida fared better than many other large states due to these efforts (115).

In February and March 2020, DOH and ACHA teams visited more than 4,000 nursing homes and assisted living facilities to provide education about COVID-19 infection control (116). On March 11, 2020, all visitation to these facilities was suspended. On March 20, 2020, Florida was one of the first states, if not the first, to recommend that all staff members wear face masks (117). DOH established a call center for facilities needing assistance, and joint DOH and AHCA strike teams visited facilities when there was a COVID-19 case (118). DEM and ACHA established COVID-19-only nursing homes (119) or COVID-19 isolation areas in nursing homes.

*Preparing hospitals and healthcare systems* for the pandemic began in mid-January 2020. Florida has more than 68,000 licensed hospital beds. Helping hospitals prepare for the pandemic, a series of weekly or twice weekly scheduled calls coordinated by the Florida Hospital Association involving DOH and ACHA started on February 3, 2020 (120).

To monitor hospital bed capacity, the ACHA activated the Emergency Status System (ESS) (121), by which hospitals electronically report information daily. Hospitalization, intensive care utilization, and ventilator accessibility were reported to the state and the Center for Medicare and Medicaid Services (CMS). After a drop in cases, the ESS system stopped collecting and publicly reporting data in the spring of 2021.

*Stopping the introduction of COVID-19 into the state* was part of the initial Florida pandemic control strategy. Following CDC travel advisory recommendations on March 9, 2020, the DOH advised people from countries and cruises with CDC level 2 and 3 COVID-19-related travel advisories to monitor their symptoms, avoid public places, and avoid being around people 65 years or with underlying medical conditions.

At large airports in the state, the DOH and the National Guard personnel screened passengers upon arrival and gave instructions for self-isolation (122). Roadblocks were set up along Florida highways at entry points to the state to identify those from areas of outbreaks, conduct health screenings, and require self-isolation (123). When Florida case counts rose in June 2020, these measures were discontinued as not viewed to be effective (124).

### K-12 schools and universities

After the closure of schools nationally to in-person education in March 2020, the adverse impact of school closures became increasingly apparent. The American Academy of Pediatrics and other organizations thus advocated for the safe return to school of children for the start of the 2020 school year (125). Besides educational and social concerns, the impact on the workforce related to school closures was considerable. Parents or guardians could not return to work while their children were at home, coupled with a lack of access to daycare. This issue also factored in the push to reopen schools for in-person education.

On July 6, 2020, Commissioner of Education Richard Corcoran issued an order requiring all publicly funded schools in the state to offer in-person instruction 5 days per week (38). The federal government provided extensive funding to the state and local school districts to support COVID-19 control measures in schools via the Elementary and Secondary School Emergency Relief (ESSER) Fund (122), with Florida receiving more than $650 million initially. School districts had the statutory authority to determine their mitigation strategies. These protective strategies varied among counties and school districts; some schools gave parents the option of face-to-face or remote learning, while in some communities, only face-to-face learning was available (126).

In preparation for the return to school, the CDC developed guidelines for the return to the classroom. However, the release of these guidelines was delayed until September 15, 2020, after children returned to schools in Florida and many other states (42, 127). In advance of CDC guidance, DOH and the Florida Department of Education (DOE) implemented numerous practices to support the safe return to school (39). Testing capacity at state laboratories was augmented so there would be a 24-h turnaround of tests, procedures for screening students on arrival to school for COVID-19 symptoms were implemented, schools had contact tracing teams, individuals with COVID-19 or exposure were identified and excluded from school per CDC guidelines (128), improved ventilation of schools took place, and school attendance was monitored. DOH developed an internal dashboard and report to monitor all schools in the state for cases of COVID-19, which were reviewed daily (129). When exposures resulted in a classroom being quarantined or other potential significant exposure events, data and response actions were reviewed centrally at the DOH.

When students returned to school in August 2020, community levels of COVID-19 were high in most of the state and in the range the CDC recommended against face-to-face education (130). The return to in-person instruction went well, as among 6,400 public K-12 schools in the state, 11% had outbreaks that could be traced to school or school-related activities (131).

Under the guidance of the Board of Governors, state universities resumed face-to-face education in August 2020 (132). Federal and state funding was also available to support these activities. COVID-19 control practices involved using facemasks, improving ventilation, COVID-19 testing, university-based contact tracing teams, and residential arrangements for individuals with COVID-19 or directly exposed to those with COVID-19 to isolate them. As in the K-12 setting, few outbreaks could be linked to the academic setting. Instead, outbreaks were most commonly related to non-classroom social activities.

The academic year was modified so students would not have traditional breaks when they would travel home and potentially reintroduce COVID-19 upon return (133). Thus, face-to-face education ended with the Thanksgiving holidays of 2020, and spring breaks were canceled for the 2021 school term (134).

The return to school in the summer of 2021 was more contentious than the previous year, as in July 2021, the Governor prohibited school districts from issuing mask mandates, and financial penalties were assessed against school officials from districts requiring mask use (134, 135). CDC guidance at this time recommended the use of face masks in schools (136).

As in the previous year, school testing for COVID-19, contact tracing, outbreak monitoring, and a focus on improving school ventilation continued. In addition, millions of home test kits were made available to schools for distribution. In contrast to the CDC policy of 10 days, the quarantine period was shortened to 7 days. Despite prohibitions against school mask mandates, the DOH return to school rule required that parents opt out of mask-wearing (137). In September 2021, mandatory quarantining of exposed students was stopped (138).

Large events were avoided at the university level during the 2020–2021 school year, but large gatherings at sporting events took place during the 2021–2022 school year. With the return of full sports stadiums in the fall of 2021 during the Delta wave, few outbreaks were associated with these outdoor activities.

### COVID-19 vaccination

The first COVID-19 vaccines to be granted FDA emergency use authorization (EUA) status were the Pfizer/Biontech vaccine on December 12, 2020 (45), followed by the Moderna vaccine on December 18, 2020 (139). The Johnson and Johnson vaccine received EUA on February 27, 2021 (140). The COVID-19 vaccine was in limited supply for adults until April 2021. Thus, vaccine allocation was prioritized by the state.

On December 3, 2020, The CDC Advisory Committee on Immunization Practices (ACIP) and the CDC Director initially recommended vaccinating those 75 years and older, essential workers, people in nursing homes, and healthcare providers (46). Considering the considerable number of people in the state who were 65 years and older, the state deviated from ACIP recommendations (141), first allocating vaccines to those 65 years and above, those certified by their physician as having a serious underlying medical condition, those in nursing homes, and healthcare workers with direct patient exposure.

COVID-19 vaccine arrived in Florida on December 14, 2020 (94). Vaccination efforts for those 65 and older were initially challenged by demand far greater than supply, outdated patient registration systems, and long lines at vaccination sites (142). Plans were implemented to limit vaccine tourism in favor of vaccination of Florida residents (143). Retail pharmacy chains helped vaccinate nursing home residents, and DOH implemented COVID-19 vaccination programs for home-bound senior citizens (144). Showing the effectiveness of this approach, by early 2021, Florida had one of the highest vaccination rates for those 65 and older individuals in the country (77).

Focusing on health equity issues to reach minority communities, DOH and DEM worked with faith-based organizations (145), with faith-based vaccination events beginning in December 2020 (146). The state established mass vaccination sites in many areas. To reach groups with high social vulnerability, FEMA initiated several vaccination sites in the state (147).

Rates of vaccination in Florida rose steadily through mid-April 2021 when those 16 years and older were approved for the vaccine. Afterward, vaccination rates sharply declined (143) following a rise in anti-vaccine sentiment and a lack of promotion by the Governor.

Before the end of the spring term for college and university students in 2021, mass vaccination events were held at sites for higher education throughout the state (148), many of which took place at sports stadiums to accommodate a substantial number of students. On May 10, 2021, the COVID-19 vaccine became available for those 12 years and older (149), and DOH partnered with pediatric providers for vaccination events (150).

On November 2, 2021, COVID-19 vaccine became available for those 5 years and older (151). Unlike CDC recommendations, the new State Surgeon General, Dr. Ladapo, stated that healthy children should not receive COVID-19 vaccines (152). On June 18, 2022, the COVID-19 vaccine was authorized for those 6 months and older (153). Florida was the only state not to pre-order vaccines for this age group.

On October 7, 2022, Dr. Ladapo advised that young men should not be vaccinated against COVID-19 due to the risk of myocarditis (71) On September 13, 2023, Dr. Ladapo recommended that individuals younger than 65 should not receive the updated COVID-19 booster (74). On January 3, 2024, Dr. Ladapo recommended that all individuals avoid COVID-19 mRNA vaccines (75). Each of these recommendations are in stark contrast to current CDC guidelines that state that all those in eligible age groups should receive updated COVID-19 shots (154).

### The delta-variant wave

Over the pandemic, there have been five waves of different variants (155). Of these, the Delta variant was the deadliest (156). This variant first appeared in India in the winter of 2021 (157) and reached the US in the spring of 2021 (158).

Individuals vaccinated against COVID-19 were protected against the severe effects of the Delta variant (159). Thus, unvaccinated or incompletely vaccinated individuals were especially susceptible to this variant. As observed in other states, COVID-19 vaccination rates in Florida varied with political affiliation (160).

The Delta wave began in Florida in July 2021, severely affecting Jacksonville in Duval County, which had lower vaccination rates than many other large cities in Florida (161). Hospital leadership reported that 95% of individuals hospitalized and dying from the Delta variant were unvaccinated. Duval County hospital capacity was so overwhelmed that a hospital executive requested the USS Comfort hospital ship to provide local support. In other areas of the state, hospitalization rates and the need for oxygen were so severe that oxygen use for water treatment plants was curtailed (157), and some hospitals only had a few days’ supply available during the Delta wave (162).

In response, in August 2021, more than 25 sites were established by DOH and DEM so that Regeneron monoclonal antibody infusions could be given to individuals with COVID-19 to lower the risk of progression to severe disease (163). Based upon known hospitalization rates with COVID-19 and the age of individuals becoming infected, approximately 1,000 infusions are projected to prevent 50 hospitalizations. More than 130,000 individuals received monoclonal antibody treatment at an average cost of $2,100 per dose, potentially preventing 6,000 hospitalizations (164).

Although it was suggested that many individuals receiving monoclonal antibodies had breakthrough cases (165), DOH did not systematically collect health information on individuals receiving infusions. Reports from hospitals indicated that more than 95% of individuals with severe COVID-19 had not been vaccinated. During the later Omicron wave in the winter of 2022, Florida continued to promote the Regeneron monoclonal strategy (166), even when the Omicron variant was not susceptible to the Regeneron monoclonal antibody, and the FDA EUA was revoked when the Omicron variant accounted for 99% of cases (167).

Over the 5 months of the Delta wave, more than 24,000 individuals died from COVID-19 in Florida compared to 38,000 individuals over the preceding 16 months. During the Delta wave peak, Florida had one of the highest COVID-19 death rates in the country (3, 168, 169). Reflecting this impact, Florida went from 26th in death *per capita* due to COVID-19 to 10th (3). If one examines COVID-19 or excess mortality deaths *per capita* over the Delta and beyond period alone, Florida ranks even worse (168, 169). It has been calculated if there had been better COVID-19 vaccine coverage through August 31, 2021, more than 16,000 deaths in Florida during the Delta wave would have been prevented (170).

### Changes in the governor’s response to COVID-19 during the pandemic

Before the first case of COVID-19 in the state, the messages delivered by Governor DeSantis focused on transparency in COVID-19 reporting and following guidelines released by the White House Coronavirus Taskforce and the CDC, including non-pharmaceutical mitigation measures. The early stages of the pandemic response action in Florida were similar to that seen in most of the country (171).

Before the first case in the state, DOH did not release information about the number of individuals tested or aggregate test results, believing that such release of information was at variance with state statute 381 (172). On February 28, 2020 (173), however, the Governor directed DOH to release all test results to promote transparency.

With the support of the Governor, separate public health advisories were issued by me, beginning in March 2020, that emphasized social distancing and mask use (110). At this time, DOH developed a COVID-19 public-facing dashboard showing cases, hospitalizations, positivity rates, and deaths per county (112).

On March 21, 2020, President Trump promoted hydroxychloroquine (168), which the FDA was pressured to grant EUA for early in the pandemic. On April 7, 2020, Governor DeSantis announced that Florida purchased 1 million doses of hydroxychloroquine from manufacturer Teva (174). The next day he held a press conference featuring a patient ill with COVID-19 treated with hydroxychloroquine to promote its use (175). Recent data show that the use of hydroxychloroquine during COVID-19 has been linked to about 17,000 deaths (176).

In mid-March, several states and Florida counties began issuing activity restriction orders to control the outbreak (177). During February and March 2020, images of college students celebrating spring breaks in Florida were broadcast as cases began to rise, triggering criticism by the media of state tourism remaining open (178). Being one of the last states to do so, on April 1, 2020, the Florida “Safer at Home” order was issued statewide by the Governor (179).

As President Trump shunned mask use, anti-face mask sentiment rose nationally (180). On April 13, 2020, the Governor’s Press Secretary removed me from a press conference after I stated mitigation measures would be needed until the COVID-19 vaccine became available.

On April 28, 2020, Governor DeSantis met with President Trump at the White House, focusing on how well Florida did during the early part of the response (31). Governor DeSantis stated, “Everyone in the media was saying Florida was going to be like New York or Italy, and that has not happened.”

The plan to reopen Florida had input from many sectors, including business, education, healthcare, and tourism. On May 4, 2020, 64 of the 67 counties in the state went to Phase 1 of reopening. As parts of the state began to reopen, DOH could trace several COVID-19 outbreaks to bars that were not adhering to limited-capacity reopening standards. On June 26, 2020, the Director of the Department of Business and Professional Relations (DBPR) ordered the bars to close again (181).

Over the summer of 2020, as COVID-19 cases began to rise, the “One Florida-One Goal” publicity campaign was launched, emphasizing the importance of mitigation measures, including facemasks (37). This campaign coincided with the second wave of COVID-19 infections that summer. Before the 4th of July holiday, the Governor emphasized wearing a mask and avoiding closed spaces and crowded places.

On July 6, 2020, the Commissioner of Education required that all schools open for in-person education (182). In some communities, there was pushback against this policy, followed by threats that the state would withhold school district funds for noncompliance (183).

In the summer of 2020, as part of the national discussion to downplay the effects of the virus, there was an attempt to show that deaths attributed to COVID-19 were not caused by the virus (184). There were incorrect claims that accident-related cases were being attributed to COVID-19 (185). A reporter was given access by the Governor’s office to the death certificates of individuals who died from COVID-19. The reporter claimed that many COVID-19-related deaths were falsely classified as such (186).

In mid-August, DOH was asked by the Governor’s office to remove the mention of masks and related infographics from the website.

A newly appointed member of the President Trump White House Coronavirus Task force, Dr. Scott Atlas, came to Florida for press conferences on August 31, 2020, to support reopening schools. He emphasized that the best way to protect older adults was to have younger individuals get COVID-19 and develop herd immunity (41).

On September 24, 2020, the Governor hosted a round table discussion with individuals who originated the Great Barrington Declaration (43). These individuals focused on the low risk that COVID-19 posed to school-age children and college students, commenting on the harm associated with in-person school closures. They advocated that the best way to protect older and at-risk individuals from COVID-19 was to have younger individuals get COVID-19, resulting in population immunity, the “let it rip” philosophy, and minimizing the effectiveness of masks (187). On September 25, 2020, local municipalities were prohibited from issuing fines to individuals not wearing masks (188).

Similar to calls issued by President Trump to reduce testing for COVID-19 (185), so there would be fewer cases diagnosed, Governor DeSantis made similar requests to DEM (189). Testing was not reduced by either DOH or DEM.

The Governor was initially enthusiastic about COVID-19 vaccines (190). The Governor signed the receipt of the shipment for the first COVID-19 vaccines to arrive in Florida (94). From December 2020 through March 2021, the Governor had numerous press conferences and public events promoting vaccination.

In April 2021, President Biden became more active in advocating for COVID-19 vaccination (191), setting a 70% national vaccination rate goal (192) by the 4th of July, 2021. After vaccine promotion comments by President Biden, Governor DeSantis, and other Republican Governors reduced or stopped promoting COVID-19 vaccination (193). There was also growing sentiment among conservatives that it was better to develop immunity to COVID-19 from infection than from vaccination (194). Whereas, COVID-19 infections can confer immunity to the virus, the degree of protection is more variable than immunity conferred following vaccination, and the risk of severe disease from natural infection outweighs the risk of vaccination (195, 196).

With the significant impact of the Delta-variant wave in July 2021, Governor DeSantis briefly resumed pro-vaccine comments (197). On September 13, 2021, he failed to correct false information from a co-speaker at a press conference stating that vaccines alter genetic material (198).

In June 2021, the DOH was instructed to replace daily COVID-19 reports with weekly reports that were initially released early Friday afternoon, and then later after 5 PM (199). This shift was to minimize COVID-19 being in the public eye.

The collapse of the condominium tower in Surfside (200) also impacted the COVID-19 response. Out of respect for this tragedy, the DOH was instructed by the to withhold all COVID-19-related messaging. This pause lasted approximately 1 month as the Delta variant wave was appearing. This pause delayed the release of a DOH-sponsored vaccine promotion campaign that had been proposed in March 2021 and was finally released in the summer of 2021 (201).

When the Delta virus hit (187), the focus was on Regeneron monoclonal antibody infusions (202). The Governor attributed the impact of the summer Delta surge to seasonality (190), not human factors.

The ESS, which provided a public snapshot of COVID-19 hospitalizations, was operational over the first half of the pandemic until cases fell in the winter of 2021. During the Delta wave, hospital executives requested that it be reactivated, but this request was denied.

In the summer of 2020, local school districts and counties determined COVID-19 policies. On July 30, 2021, the Governor overrode local guidelines and prohibited mask mandates in K-12 schools (135). In August 2021, the Governor began focusing on how respiratory syncytial virus (RSV), was more of a threat than COVID-19 to children (203).

On March 18, 2021, the Governor hosted a round table discussion with originators of the Great Barrington Declaration, in which the use of face masks by children was discredited. The next day, YouTube removed the video of the press conference saying that it contradicted expert opinion (204).

On April 2, 2021, the Governor prohibited businesses from having vaccine passports for business entry or COVID-19 vaccine requirements (205). DOH was directed to investigate circumstances where companies inquired about vaccine status for entry. DOH was charged with investigating these violations, with a penalty of $5,000 per event per person (206).

On July 13, 2021, Norwegian Cruise Lines, which had a vaccine requirement for passengers, sued me as representative of the state of Florida over this issue (207). A federal judge ruled that the cruise industry was entitled to require COVID-19 vaccination for passengers and staff (208).

On July 13, 2021, As COVID-19 cases in Florida reached the highest in the nation, the Governor DeSantis campaign team rolled out “Do not Fauci My Florida” merchandize (58).

With the rise of COVID-19 cases during the Delta variant surge in Florida, on August 3, 2021, President Biden admonished Governor DeSantis to “get out of the way,” of the state COVID-19 response (209). This episode triggered pushback by Governor DeSantis against President Biden’s COVID-19 policies (210).

On September 4, 2021, President Biden called for vaccine mandates for businesses with 100 employees or more and health care workers. On October 28, 2021, Governor DeSantis announced a lawsuit against the vaccine mandates (63).

On November 18, 2021, Employers were prohibited from requiring employees to be vaccinated against COVID-19 (135). When the Supreme Court upheld the Biden administration’s requirement that healthcare workers be vaccinated on January 14, 2022, Governor DeSantis stated that Florida would not enforce the ruling (211).

At a press conference on March 2, 2022, the Governor chided high school students for wearing masks, calling their use “political theater.” (212).

On December 13, 2022, the Governor empowered a grand jury to investigate vaccine manufacturers for COVID-19 (198). A partisan Public Health Integrity Committee, constituted by supporters of the Great Barrington Declaration, was also commissioned to assess the response to the pandemic (72, 73).

On May 11, 2023, the legislation banning COVID-19-related mandates was enacted (213).

On November 2, 2021, COVID-19 vaccine became available for those 5 years and older (151). In contrast to CDC recommendations, the new State Surgeon General, Dr. Ladapo, stated that healthy children should not receive COVID-19 vaccines (152).

On June 18, 2022, COVID-19 vaccine was authorized for those 6 months of age and older (153). Florida was the only state not to pre-order vaccines for this age group.

On October 7, 2022, Dr. Ladapo advised that young men should not be vaccinated against COVID-19 due to the risk of myocarditis (71).

On September 13, 2023, Dr. Ladapo and Governor DeSantis recommended that individuals younger than 65 should not receive the updated COVID-19 booster (74).

On November 1, 2023, after announcing that individuals younger than 65 should not receive the updated COVID-19 booster (214), Dr. Ladapo campaigned with Gov. DeSantis in New Hampshire to promote medical freedom (215).

.On January 3, 2024, Dr. Ladapo recommended that all individuals avoid COVID-19 mRNA vaccines (75).

### Synopsis

As the COVID-19 death toll passes 93,000 in Florida, it is essential to assess what worked well in the response and what did not. The answer to these questions lies in examining the different strategies and actions over the first 16 months of the pandemic, during which Florida had one of the lower mortality rates in the US despite being one of the most medically vulnerable states, to being one of the most severely impacted states during the Delta variant wave and beyond.

Over the first phase of the pandemic, there was a strong focus on mitigation and vaccination measures, with emphasis directed toward protecting older individuals who were most vulnerable to the virus. We also observed that before the availability of COVID-19 vaccines, public health measures facilitated the successful phased re-opening of communities and businesses after the April 2020 lockdown. We also observed that public health measures supported the successful reopening of K-12 schools and institutions of higher education at a time the country was divided on the school reopening issue, and many schools did not open nationally.

Over the first 6 months after vaccines first became available, there was considerable enthusiasm for COVID-19 vaccination, especially among older people, contributing to the low state mortality rates. Yet, with the rise of COVID-19 misinformation, anti-vaccine sentiment, and waning political support for COVID-19 vaccines by the Governor, vaccination rates fell, resulting in population vulnerability. Thus, when the severe Delta variant wave hit Florida in the summer of 2021, cases of severe COVID-19 spiked in younger and unvaccinated individuals. Continuing this trend, death rates in Florida have been among the highest in the country during recent waves of Omicron variants.

There are lessons to be learned not just for Florida but for the country in examining the response to and the impact of COVID-19 on the state. Rather than the partisan Public Health Integrity Committee, commissioned by Governor DeSantis to assess the response to the pandemic (73), the state would be much better served by the bipartisan panel with scientific expertise. Such was the model implemented last year, by the Republican Governor of Indiana, who convened a bipartisan commission with respected public health experts to assess health issues in the state, including COVID-19 and the public health response (216). Alternatively, medical schools and schools of public health in Florida could collectively perform the type of independent, rigorous analysis they are capable of or at least begin to advocate for such.

For now, we can see the clear benefit of COVID-19 mitigation and vaccination strategies over the first phase of the pandemic in Florida. We can also see the consequences of abandoning core mitigation strategies and stopping the promotion of protective vaccines, looking at how Floridia has been affected by severe COVID-19 ever since.

## Author contributions

SR: Conceptualization, Writing – original draft, Writing – review & editing.
